# Does intermittent pneumatic compression reduce the risk of post stroke deep vein thrombosis? The CLOTS 3 trial: study protocol for a randomized controlled trial

**DOI:** 10.1186/1745-6215-13-26

**Published:** 2012-03-08

**Authors:** Martin Dennis, Peter Sandercock, John Reid, Catriona Graham, John Forbes

**Affiliations:** 1 Division of Clinical neurosciences, University of Edinburgh Western General Hospital, Edinburgh EH4 2XU, UK; 2 Borders General Hospital, Melrose, Roxburghshire TD6 9BS, UK; 3 Epidemiology and Statistics Core, Wellcome Trust Clinical Research Facility, University of Edinburgh Western General Hospital, Edinburgh EH4 2XU, UK; 4 Centre for Population Health Sciences, The University of Edinburgh Medical School, Teviot Place, Edinburgh EH8 9AG, UK

**Keywords:** Stroke, Deep vein thrombosis, Prevention, Intermittent Pneumatic Compression, Complications

## Abstract

**Abstract:**

**Background**

Approximately 80,000 patients each year are admitted to UK hospitals with an acute stroke and are immobile. At least 10% will develop a proximal Deep Vein Thrombosis in the first month and 1.5% a pulmonary embolus. Although hydration, antiplatelet treatment and early mobilisation may reduce the risk of deep vein thrombosis, there are currently no preventive strategies which have been clearly shown to be both effective and safe. Anticoagulation increases the risks of bleeding and compression stockings are ineffective. Systematic reviews of small randomized trials of intermittent pneumatic compression have shown that this reduces the risk of deep vein thrombosis in patients undergoing surgery, but that there are few data concerning its use after stroke. The CLOTS trial 3 aims to determine whether, compared with best medical care, best medical care plus intermittent pneumatic compression in immobile stroke patients reduces the risk of proximal deep vein thrombosis.

**Methods/Design**

CLOTS Trial 3 is a parallel group multicentre trial; with centralized randomisation (minimisation) to ensure allocation concealment. Over 80 centres in the UK will recruit 2800 immobile stroke patients within the first 3 days of their hospital admission. Patients will be allocated to best medical care or best medical care plus intermittent pneumatic compression. Ultrasonographers will perform a Compression Duplex Ultrasound Scan to detect deep vein thrombosis in each treatment group at about 7-10 days and 25-30 days. The primary outcome cluster includes symptomatic or asymptomatic deep vein thrombosis in the popliteal or femoral veins detected on either scan. Patients are then followed up by postal or telephone questionnaire at 6 months from randomisation to detect later symptomatic deep vein thrombosis and pulmonary emboli and to establish their functional outcome (Oxford handicap scale) and quality of life (EQ5D-3 L). The ultrasonographers performing the scans are blinded to treatment allocation, whereas the patients and caregivers are not. The trial has 90% power to detect a 4% absolute difference in risk of the primary outcome and includes a health economic analysis.

**Discussion**

The trial started recruitment in Dec 2008 and will complete recruitment during 2012. It will report results in 2013.

**Trial registration number:**

ISRCTN: ISRCTN93529999

## Background

A House of Commons Health Committee highlighted in 2005 the very large number of patients dying in UK hospitals from venous thromboembolism and called for more effective prophylaxis [1]. Studies reported over the last 30 years have shown that deep vein thrombosis (DVT) is particularly common in patients with a recent stroke. Patients with significant weakness of the leg and who are immobile appear to be at greatest risk. Studies with magnetic resonance imaging demonstrated DVT in 40% of stroke patients within the first three weeks, and above knee DVT in 18% [2]. Studies using less sensitive screening techniques, such as compression duplex ultrasound (CDU), demonstrate a lower frequency of above knee DVT of about 10% although the types of patients included and the duration and timing of follow up influences the estimates [3]. Clinically apparent DVT confirmed on investigation is less common but DVTs may not be recognised and may still cause important complications. Pulmonary embolism (PE) is an important cause of preventable death after stroke [4]. Clinically evident PE has been variably estimated to affect one to 16% of patients in prospective trials [5] and three to 30% in observational studies [6]. In the CLOTS Trial 1 about 5% of patients developed symptomatic DVT and 1.5% had a confirmed PE in the first month after stroke [3]. The rate of PE is likely to be underestimated because they are not routinely screened for, and autopsies are rarely performed. Many patients who have pneumonia or unexplained fever may actually have pulmonary emboli. Fifty percent of patients who die following an acute stroke showed evidence of PE on autopsy [7]. Studies, like the CLOTS Trial 1, which screen for DVT may under-estimate the clinical importance of venous thromboembolism because patients are usually treated whilst still asymptomatic so their risk of developing symptomatic DVT and pulmonary embolism is reduced.

Traditionally a number of interventions have been used to reduce the risk of DVT. These include:

Anticoagulants: A Cochrane review showed that both low and medium dose subcutaneous heparin reduce the risk of DVT, and probably PE, in patients with acute ischaemic stroke [5]. However, evidence from the International Stroke Trial [8] showed that even low dose heparin (5,000 units twice daily) is associated with a significant excess of symptomatic intracranial and extracranial bleeds which offsets any other advantages heparin may have on recurrent ischaemic stroke and fatal PE. The PREVAIL Trial compared the effectiveness, and safety of standard prophylactic heparin with low molecular weight heparin (LMWH) [9]. It demonstrated that LMWH was probably more effective than standard heparin in reducing the risk of mainly asymptomatic DVTs but it was not powered to identify clinically significant differences in risk. More importantly it does not, despite reports to the contrary, provide any evidence that routine heparin or LMWH use is associated with net benefit - it had no "avoid any heparin" control group [10].

Graduated Compression Stockings (GCS): Although, GCS seem to reduce the risk of DVT in patients undergoing surgery [11,12], the CLOTS Trial 1 showed that thigh-length GCS were not associated with a clinically useful reduction in the risk of post stroke DVT (absolute reduction in risk of proximal DVT = 0.5% (95%CI -1.9 to 2.9))[3,13].

Intermittent Pneumatic Compression (IPC): This comprises a pair of inflatable sleeves which are wrapped around the legs and are secured by Velcro™. They are attached via flexible tubing to a small bedside electric pump. The sleeves may be short (or below knee), wrapping around just the lower leg, or long (thigh length) to wrap around the thigh as well. They are inflated one side at a time to compress the legs at intervals. Some types inflate sequentially, first around the lower leg and then the upper, to "milk" the blood from the leg and increase venous flow. The frequency of inflation can be fixed, or in more sophisticated systems varies depending upon the rate of venous refill. IPC is thought to reduce the risk of venous thrombosis by:

• increasing the flow of venous blood through the deep veins of the leg to reduce the likelihood of thrombosis.

• stimulating release of intrinsic fibrinolytic substances.

IPC has mainly been used in surgical patients during and immediately after operations. A systematic review identified 22 randomised trials of IPC, which included a total of 2779 patients. Use of IPC was associated with a 64% reduction in the odds of DVT (*p *< 0.00001) [12]. This review concluded that a priority for future research was trials of "prevention of venous thromboembolism with mechanical methods among high-risk medical patients (such as those with stroke)".

A Cochrane review [13] of the effectiveness of physical means of reducing the risk of venous thromboembolism after stroke identified only two small trials of IPC including just 177 patients in total. IPC was associated with a non-significant trend towards a lower risk of DVTs (OR 0.45, 95% CI 0.19 to 1.10) with no evidence of an effect on deaths (OR 1.04, 95% CI 0.37 to 2.89).

Thus, the available evidence confirms that after stroke, even applying current prophylactic strategies, the risk of venous thromboembolism is substantial. The available data suggest that IPC is a promising, but unproven and rarely used intervention. The CLOTS trial 3 aims to:

1. Establish whether the routine application of IPC to the legs of immobile stroke patients reduces their risk of DVT and PE.

2. Determine whether IPC adds to the benefits of routine care which often includes good hydration, early use of aspirin and mobilisation.

3. Quantify any risks of IPC when applied to stroke patients.

4. Estimate the cost-effectiveness of IPC which will help health service planners decide whether IPC should be offered routinely in the UK NHS.

5. Provide robust estimates of the effectiveness of IPC in stroke patients which might be extrapolated to other groups of medical (rather than surgical) patients at high risk of venous thromboembolism.

## Methods/Design

### Design overview

**Figure 1:**
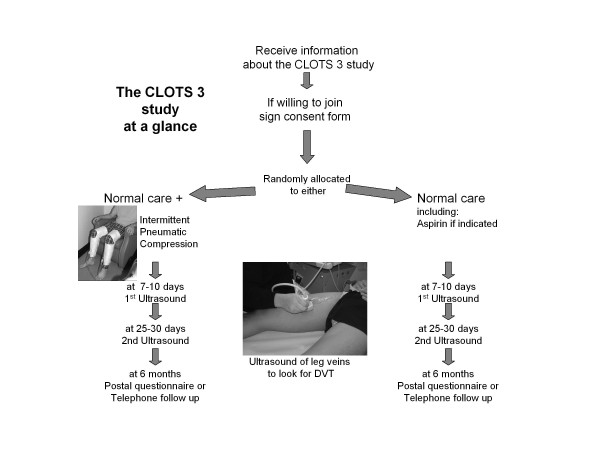
**Simplified trial flowchart**.

CLOTS trial 3 is a multicentre, parallel group trial with a centralized randomisation system to allocate treatment with a 1:1 ratio, and which ensures allocation concealment (see Figure 1). Its methods are very similar to those of CLOTS trial 1 & 2 [3,14]. We aim to blind the ultrasonographers, who carry out the scans to detect DVTs, but are unable to blind the patients and their caregivers to allocation group because of the nature of the intervention. The Multicentre Research Ethics Committees in the UK and the local ethics committees in all contributing centres approve our protocol. We obtain written informed consent from all patients, or for patients lacking mental capacity, from the patients' personal legal representatives. The trial is registered (ISRCTN93529999).

### Setting and participants

Our collaborators in more than 80 centres in the UK aim to enrol at least 2800 patients. To participate, hospitals have to have: a local principal investigator who takes responsibility for the trial governance; a well organised inpatient stroke service; nursing staff trained in the use of IPC; and a diagnostic ultrasound department which routinely performs CDU.

## Inclusion criteria

• Any patient admitted to hospital within 3 days of a clinical stroke fulfilling the WHO criteria.

and

• Who is not able to get up from a chair/out of bed and walk to the toilet without the help of another person

Patients can be randomised from Day 0 (day of admission) to Day 3 of hospital admission. If a patient has a stroke during a hospital admission they are eligible until Day 3 from the stroke onset (Day 0). Stroke should be the most likely clinical diagnosis but a visible infarction does not have to be seen on a brain scan.

## Exclusion criteria

• Patients under 16 year of age

• Patients with stroke due to subarachnoid haemorrhage.

• Patients who, in the opinion of the responsible clinician/nurse, are unlikely to benefit from IPC - for instance, this would include patients who are expected to mobilise within the next day.

• Patients with contraindications for the use of IPC. These include:

○ patients with local leg conditions in which the IPC sleeves would interfere such as dermatitis,

○ patients with severe arteriosclerosis or as indicated by absence of pedal pulses or history of definite intermittent claudication.

○ patients who have massive leg oedema or pulmonary oedema from congestive heart failure.

• Patients who already have swelling or other signs of an existing DVT. Such patients may be recruited once a DVT has been excluded by normal D Dimers or CDU. There is a concern that if one applied IPC to patients who may already have a DVT it may displace the thrombus and increase the risk of PE. However, this potential risk has not been documented in the RCTs so far. And we have not identified any case reports where there is convincing evidence that this has occurred.

Inclusion in another research study, including another randomised controlled trial, does not automatically exclude a patient from participating in CLOTS 3. As long as inclusion in the other study would not confound the results of CLOTS 3, co-enrolment is permissible. Also, local researchers must avoid overburdening patients. Patients should not be co-enrolled in another research study which aims to test an intervention which aims to reduce the risk of venous thromboembolism.

## Consent

The patient, or their legal representative, are approached by a member of the clinical team looking after that patient to ascertain their interest in participating in the CLOTS 3 trial or to obtain their permission to pass their details onto any research staff involved. Written informed consent is sought where possible. If this is not possible the randomising clinician or nurse can gain witnessed verbal consent. Patients or legal representatives are given enough time to consider the trial fully and ask any questions they may have about the implications of the trial.

### Randomisation and interventions

Having obtained consent, the clinician enters the patient's baseline data into our computerised central randomisation service via a secure web interface or a touch-tone telephone system. We encourage clinicians to enrol patients as early as possible since prophylaxis for DVT will have greater effect if started early. Once the computer program has checked these baseline data for completeness and consistency, it generates that patient's treatment allocation - either "best medical care plus IPC" or "best medical care alone". The system applies a minimisation program to achieve balance for four prognostic factors:

• Delay since stroke onset. (Day 0 or 1 vs. Day 2-7)

• Stroke severity (using a validated prognostic model [15] which includes the following factors; age, pre stroke dependency in activities of daily living, living with another person prior to stroke, able to talk and orientated in time, place and person, and able to lift both arms to horizontal position against gravity).

• Severity of leg paresis (able or not to lift leg off the bed)

• Use of heparin, warfarin or thrombolysis at the time of enrolment

Because simple minimisation can theoretically lead to alternation of treatment allocation, our system also incorporates a degree of random allocation - i.e. it allocates patients to the treatment group that minimises the difference between the groups with a probability of 0.8 rather than 1.0[16]. This helps to guarantee allocation concealment.

If allocated IPC, nursing staff size, fit and apply the sleeves, based on the manufacturers (Covidien,MA, USA) instructions, to both legs as soon as possible after the randomisation phone call. The IPC sleeves should be worn both day and night, whilst the patient is in the bed or chair for 30 days from randomisation OR until a second screening CDU has been performed (if after 30 days), OR it may be removed earlier if the:

• patient is independently mobile around the ward (i.e. can get up from a chair/out of bed and walk to the toilet without the help of another person).

• patient is discharged from the participating hospital. If the patient is transferred to a rehabilitation unit where it is practical to continue the IPC and monitor its use appropriately then IPC should be continued until independently mobile or the patient declines to continue or an adverse effect of IPC occurs. If IPC cannot be continued after transfer to a rehabilitation unit a discharge form should be completed at the time of transfer to the rehabilitation unit.

• patient declines to continue to have IPC applied.

• healthcare staff identify any adverse effect of the IPC (such as pressure ulcers, falls due to the IPC) which they judge make continued application of the IPC unwise.

If the IPC is removed for any other reason e.g. checking the legs, bathing, screening CDU, then the IPC should be replaced as soon as possible. If the sleeves become soiled they should be replaced with new sleeves as soon as possible.

Our recruitment coordinator and representatives of Covidien provide onsite training to nursing staff in the correct sizing, fitting and monitoring of IPC. This is supplemented by a training video and web-based training. We ask nursing staff to record their use of IPC on the medication chart to increase compliance and aid monitoring. We stipulate that both treatment groups should receive the same medical care that could include, depending on local protocols, early mobilisation, hydration, antiplatelet or anticoagulant drugs. The local coordinator extracts information from the medication charts on the compliance with IPC and use of antiplatelet and anticoagulant drugs during the admission and records this on our hospital discharge form, so we can check that these aspects of medical care are used equally in the treatment groups.

## Outcomes and follow-up

### Primary outcome

The primary outcome is the occurrence of either: a symptomatic or asymptomatic DVT in the popliteal or femoral veins (detected on the first or second CDU performed as part of the trial protocol) or a symptomatic DVT in the popliteal or femoral veins, confirmed on imaging (either CDU or venography) within 30 days of randomisation. We focus on proximal DVTs because they are much more reliably detected by ultrasound and are generally regarded as clinically more important [17,18].

### Secondary outcomes

In hospital or within 30 days:

• Death within 30 days

• Presence of definite or probable DVT in the popliteal or femoral veins detected on a screening CDU scan which had not been suspected clinically before the scan (see below)

• Definite (i.e. excluding probable DVTs) symptomatic or asymptomatic DVT in the popliteal or femoral veins detected on either a CDU scan or contrast venography or MRI direct thrombus imaging within 30 days of randomisation;,

• Any definite or probable symptomatic or asymptomatic DVT (i.e. including DVTs which only involve the calf veins),

• Confirmed fatal or non-fatal PE,

• Adherence to allocated treatment

• Adverse events related to IPC within 30 days of randomisation.

• At six months:

• death from any cause

• any confirmed symptomatic or asymptomatic DVT or PE occurring between randomisation and final follow up

• any symptomatic DVT or PE occurring between randomisation and final follow up

• place of residence,

• post DVT syndrome,

• functional status, three simple questions [19] and Oxford handicap scale [20],

• health related quality of life (EuroQol)[21].

## Adverse events

Stroke is a serious medical condition. About 20% of hospitalized patients would be expected to die. Serious medical complications are common. CLOTS 3 is evaluating IPC, a non-drug intervention which has a CE mark and has been approved for the purpose of reducing the risks of venous thromboembolism. The risks associated with IPC and participation in the trial are very small and generally predictable e.g. skins problems on legs, falls resulting in injury. It should be relatively straightforward to attribute any serious adverse event to the IPC. In this trial we therefore do not require routine reporting of any adverse events since this is unlikely to be informative and places an unnecessary burden on the local researchers which would compromise the practicality of the trial. We do require prompt reporting of primary and secondary outcomes on the radiology report form (within 2 working days), discharge form (within 10 working days), General Practitioner questionnaire and hospital follow up forms.

The following should be reported on the radiology report form, discharge form or General Practitioner questionnaire (if patient has been discharged) or hospital 6 month follow up form (if the patient is still in hospital):

• Any confirmed DVT

• Any confirmed pulmonary embolus

• Any fall associated with significant injury occurring within 30 days of enrollment (when IPC might still be applied) - an injury requiring investigation, or specific treatment or which prolongs hospitalisation or leads to death, temporary or permanent disability.

• Any damage to the skin of the legs including necrosis, ulcers occurring within 30 days of enrollment

• Reasons for prematurely stopping the IPC

The following are expected complications of stroke and its co-morbidities and do not need to be reported as Adverse Events:

• Deaths - these should be reported as outcome events on the discharge or 6 month follow up forms

• Infections other than those affecting the skin of the legs

• Further vascular events (including recurrent strokes, myocardial infarction, bowel ischaemia)

• Cardiac, renal or liver problems

• Epileptic seizures

• Spasticity or contractures

• Painful shoulder and other joint problems

• Mood disturbances

Any other adverse events which the investigator feels may be due to either the treatment or participation in the trial should be reported within 10 working days to the coordinating centre. A serious adverse event (i.e. one resulting in death, is life threatening, results in significant disability or incapacity or prolongation of hospitalisation) should be reported immediately on a Serious Adverse Events Form on line or by FAX. Serious Adverse Events attributed to the trial treatment or participation in the trial will be reported to the Data Monitoring Committee (DMC), Trial sponsors and ethics committees.

## Follow up

### Detection of DVT

Patients should have a CDU of the veins in both legs between Day 7 and Day 10 and usually between Day 25 and 30. We define minimum acceptable technical standards for ultrasound equipment and stipulate that the trial ultrasonographers should have performed CDU to diagnose DVTs as part of a clinical service. We asked them to visualise the popliteal and femoral veins in both legs but do not insist that they routinely visualise the six deep veins in the calf since detecting thrombosis in these is far less reliable. We obtain a hard copy of positive scans to enable our trial radiologist (JR), who is blinded to group allocation, to verify each primary outcome. We do not perform central verification of negative scans because, with ultrasound techniques, meaningful verification of static images is difficult. If the second ultrasound is delayed to more than 30 days and shows a popliteal or femoral DVT, it is included in the primary outcome. However, we do not include a proximal DVT in our primary outcome which only comes to attention because of symptoms starting more than 30 days after enrollment because this might introduce bias.

Where the randomising person judges that it is likely to be impractical to perform a CDU between Days 25 and 30, they may, prior to randomisation, stipulate that a CDU will only be performed between Days 7 and 10. This might be the case if the patient is likely to be discharged home to another region or transferred to a rehabilitation facility that does not have use of CDU facilities and is remote from the randomising centre.

If a definite above knee DVT is detected on the first screening CDU i.e. the patient has our primary outcome then the second screening CDU is no longer required.

IPC should be removed completely before the patient leaves the ward to have the CDU to ensure optimal blinding of the primary outcome measure. The CDU operator is asked to guess which treatment group the patient is in prior to the examination to estimate the effectiveness of blinding. In those patients allocated IPC it should immediately be re-applied on return to the ward after the screening CDU.

### In hospital follow up

The local coordinator completes a discharge form for all randomised patients on discharge from the randomising centre or in the event of earlier death. We can not blind the local coordinator to group allocation. If a patient is transferred to a rehabilitation unit on a different site to the randomising centre, and it is impractical to continue the allocated treatment or its monitoring whilst the patient is in that unit a discharge form is completed on transfer to that unit.

The data collected at hospital discharge includes:

• Use of heparin, warfarin, and antiplatelet drugs during admission to monitor the components of routine care and to ensure equal use in the two treatment arms. However, an imbalance of heparin (or similar) and warfarin may occur if IPC is effective since more patients in the control arm will receive these drugs to treat the excess of venous thromboembolism. The indication for their use is therefore recorded.

• Use of full length or below knee graduated compression stockings to monitor the components of routine care and to ensure equal use in the two treatment arms.

• Timing of initiation of IPC and adherence to treatment allocation and use of IPC.

• Any clinical DVT or PE requiring treatment.

• Any complications of treatments in particular skin problems with legs, falls resulting in injuries occurring within the first 30 days after randomisation.

The discharge form includes checkboxes to record these secondary outcomes and adverse events. The date of occurrence of any secondary outcome is recorded along with a free text description of the problem. The chief investigator (MD) reviews these data centrally and codes the events as far as possible blind to the group allocation.

### Later outcomes

The coordinating centre telephones and sends a postal questionnaire to the General Practitioners of all patients who survive to discharge from hospital about 24 weeks after randomisation. This establishes that the patient is alive prior to sending out a follow-up form and ascertain whether they have had any DVT or PE since discharge from the randomising centre.

### Six month follow up

The Co-ordinating Centre sends a postal questionnaire (and one postal reminder and then a telephone follow up for non responders) to those surviving patients who have been discharged. The six month questionnaire aims to establish:

• the place of residence (own home, with relatives, residential care or nursing home) [as a guide to resource use]

• their functional status

• their current antithrombotic medication regimen

• presence of leg swelling, ulcers which might reflect post DVT syndrome

If the patient is still in hospital when the six month follow-up is due, the randomising clinician/nurse will be sent a six month "in hospital" follow-up form which should be completed with the patient. We check data centrally for completeness and consistency and sent monthly reports to each centre with data queries.

## Management of DVT in the trial

If the clinician is satisfied that the patients has a proximal DVT (with or without a confirmatory venogram) the patient should be anticoagulated using subcutaneous heparin/LMWH according to local protocols as long as there is no contraindication. If only calf vein thrombus is detected (by screening CDU and/or venography), the responsible clinician may anticoagulate the patient according to local protocols or alternatively arrange a follow up CDU approximately seven days later to identify any propagation into the popliteal vein. If definite popliteal or femoral vein thrombus is detected the patient should be anticoagulated unless contraindicated. If a patient develops symptoms or signs suggestive of DVT during their admission they should be investigated by either CDU and/or venography or MRI direct thrombus imaging and treated according to local protocols if the diagnosis is confirmed. Use of heparin, LMWH and Warfarin to treat DVT and/or PE during admission is recorded on the discharge form. Continued use of IPC in such patients is at the discretion of the responsible clinician.

## Sample size

We originally planned to enrol at least 2000 patients. This aimed to give the Trial > 90% power (alpha 0.05) to identify an absolute reduction of risk of our primary outcome of 4% (about 10% to 6%). The frequency of our primary outcome was estimated from the CLOTS trials 1 and 2. We aimed to enrol at least 75% of patients on Days 0-2 after stroke onset. If the proportion enrolled after Day 2 exceeds 25% of the total then the Trial Steering Committee (TSC) can consider raising the overall target. This should help ensure that we do not miss a real treatment effect because of delays in recruitment.

In October 2010, the frequency of the primary outcome in both groups combined was 12.2%, rather than the anticipated 8%. The TSC therefore revised the sample size to 2800 to ensure that the trial maintained power to detect a 4% absolute difference in proximal DVT (i.e. a reduction from 14% in the best medical care group to 10% in the best medical care plus IPC group).

## Analyses

The trial statistician (CG) prepares analyses of the accumulating data which the independent Data Monitoring Committee (DMC) reviews in strict confidence at least once per year. No other members of the trial team, TSC or participants have access to these analyses.

A detailed analysis plan will be prepared by the members of the TSC prior to the completion of enrollment without input from the trial statistician or reference to the unblinded data and then published on the trial website. For the purposes of all primary analyses, we will retain participants in the treatment group to which they were originally assigned irrespective of the treatment they actually received. We will strive to obtain full follow-up data on every patient to allow a full intention-to-treat analysis. Inevitably, some patients will be lost to follow up. We will exclude these patients from the analyses that they have no data for, and do sensitivity analyses to see the effect of these exclusions on our conclusions. For binary outcomes (e.g. occurrence of a primary or secondary outcome OR not), outcomes will be presented as odds ratios and 95% confidence intervals, adjusted using logistic regression for the factors used in the minimisation algorithm. We will calculate absolute reductions in risk from these values. The Oxford handicap scale will be analysed in two ways - dichotomized in OHS 0-2 vs 3-6 (by logistic regression) and as an ordinal scale (by ordinal regression). The utility based on the EuroQol will be compared by t-tests if the data are Normally distributed, and using an appropriate nonparametric test otherwise.

### Preplanned subgroup analyses

Preplanned subgroup analyses include: the effect of treatment allocation on the primary outcome subdivided by key baseline variables:

• Time from stroke onset to randomisation (Day 0 or 1 vs. 2 to 7 and Day 0 to 2 vs. 3-7). Since DVT may develop very soon after stroke onset and IPC may not influence propagation of thrombus which has already started it is plausible that IPC will be more effective if started earlier after stroke.

• Paralysis of leg (complete vs. incomplete)

• Stroke severity (using a validated prognostic model [15]

• Use of heparin, warfarin or thrombolysis at the time of enrolment

Subgroup analyses will be performed by observing the change in log-likelihood when the interaction between the treatment and the subgroup is added into a logistic regression model. These analyses will be repeated but using the occurrence of our primary outcome within 14 days of randomisation (instead of 30 days).

We will perform secondary on treatment analyses to examine the extent to which non adherence to the trial intervention might explain the estimate of effect size.

### Economic analyses

Economic analysis of trial treatment effects will involve a within trial evaluation of cost effectiveness integrated into a decision-analytic model of longer run costs and health effects. The within trial analysis will be conducted on an intention-to-treat basis. The primary health endpoints will be survival times adjusted for quality of life. A standard multiplicative model will be used to estimate quality adjusted life years (QALYs) by the area under linear interpolation of the EQ5D-3 L index trajectory for each individual using survival times, the EQ5D-3 L index score at six months and a modelled baseline EQ5D -3 L index score. We will assess robustness using probabilistic sensitivity analysis of the parameters used to generate the short-run QALY estimates.

A NHS perspective will be adopted for assessing resource use and costs. Patient-specific hospital resource use will be measured using the duration of stay for the index episode following randomisation. The net direct medical cost will include the hospital stay, converted into cost estimates using NHS per-diem hospital costs, a cost estimate of IPC capital/equipment (and staffing implications) and the averted costs arising from the effects of IPC on expected DVT/PE incidence. Trial centre/region specific per-diem hospital costs will be based on NHS reference costs in England and cost information for NHS Scotland derived from the Scottish Health Service Costs resource. Probabilistic sensitivity analysis will also be used to assess the hospital cost distributions.

We will assess differences in costs and effects using econometric methods based on a copula framework that is particularly useful and straightforward when modelling joint parametric distributions. We will also summarise our cost effectiveness results within a net benefit approach using incremental net (monetary) benefit and cost-effectiveness acceptability curves.

Costs and benefits of an effective approach to preventing DVT following stroke will accrue over time. An important element of the economic analysis will be a focus on longer run outcomes using a decision-analytic model that builds on the within trial findings. The key parameters for the patient level simulation model will include expected survival, quality of life, long term complications, such as post-thrombotic syndrome, and use of health services over a 6 month to 5 year time horizon. The model will be calibrated using distributions from reported systematic reviews of survival and health-related quality of life following stroke and the long term prognosis and cost burden of DVT in the community. Monte Carlo probabilistic sensitivity analysis will be used to account for uncertainty in the cost effectiveness results based on the simulation model.

## Research governance

The Principal Investigator's (PI) in each centre is responsible for:

• Discussing the trial with medical and nursing staff who see stroke patients and ensure that they remain aware of the state of the current knowledge, the most recent trial protocol and its procedures.

• Delegating roles to those with appropriate knowledge and skills.

• Ensuring that patients admitted with stroke are considered promptly for the trial.

• Ensuring that the randomisation forms, radiology report forms and discharge forms are completed and either entered on line or sent to the coordinating centre promptly and that copies are kept in a site file and patient notes.

• Ensuring the trial is conducted in accordance with GCP and fulfils all national and local regulatory requirements.

• Ensuring that the patients' confidentiality is not breached.

• Allowing access to source data for audit and verification.

The Co-ordinating Centre is responsible for:

• Providing study materials, a 24-hour randomisation service and Helpline.

• Giving collaborators regular information about the progress of the study.

• Helping ensure complete data collection at discharge.

• Responding to any questions (e.g. from collaborators) about the trial.

• Assuring data security and quality in accordance with GCP and local guidelines.

• Ensuring trial is conducted in accordance with GCP.

## Monitoring

IPC devices carry a CE mark and are licensed for use as a prophylaxis for venous thromboembolism. In surgical practice, their use appears to be associated with a low risk of adverse effects. The trial procedures are relatively simple and place only a small burden on the patients. No significant financial inducements are being offered to collaborating centres to encourage their active participation or to reward high recruitment rates. Central follow up of all patients at about six months after enrolment provides confirmation of the trial participants identity (hence avoids the need for detailed on site source data verification of patient identity, a very resource intensive activity). After an appropriate risk assessment process, the trial management group and the trial sponsors judged that: the risks of patients being harmed by the trial interventions were small; any hazard associated with participation in the trial was very small; and, the risk of research misconduct are also small. The intensity of on-site monitoring which we undertake is based on this risk assessment. The coordinating centre monitors the completeness, internal consistency and validity of the data from all trial sites, and applies the central statistical techniques proposed by Buyse et al. [22]. From the data collected we monitor adherence to the trial protocol. Our study monitor carries out source data verification in a small random sample of patients during on site visits. If concerns arise as a result of the routine central statistical monitoring, a more detailed investigation including on-site verification of data is carried out.

The trial is jointly sponsored by NHS Lothian and the University of Edinburgh.

## Discussion

This is to our knowledge, the largest RCT of IPC ever. It will include more patients and outcome events (proximal DVTs) than all previous RCTs of IPC combined. The patients are enrolled by over 80 hospitals which hopefully will mean that our results will have good external validity. Central randomisation, mainly blinded assessment of our primary outcome, low losses to follow up and intention to treat analysis will minimise bias.

In this trial the primary outcome is an intermediate level outcome. Ideally, we would demonstrate the effect of IPC on symptomatic DVT, PE, survival and functional status. However, symptomatic events are far rarer than those identified by screening CDU and the impact on survival and functional status relatively small. Therefore, a trial would need to enrol many tens of thousands of patients to demonstrate moderate-sized, but clinically important, effects on these outcomes. We can justify the use of an intermediate outcome because of the clinical significance attributed to even asymptomatic DVT in popliteal or femoral veins which most clinicians treat with anticoagulant therapy. Also, IPC is very unlikely to have adverse effects beyond those which will be directly measured in the trial i.e. damage to skin on legs, or falls. IPC should not restrict mobilisation because it can be worn during bed to chair transfers and will be taken off when the patient is able to mobilise independently. Theoretically, IPC might influence blood pressure in the acute phase, which could in turn influence stroke outcome, by increasing venous return - however empirical studies have not demonstrated a significant effect of IPC on blood pressure. The CLOTS trial may underestimate the clinical importance of the effects of IPC because we do not systematically screen for pulmonary emboli with routine imaging. Also, because we systematically screen for asymptomatic DVT, many patients receive treatment with anticoagulants to lessen the risk of symptomatic events occurring.

In stroke patients, and those with other acute medical conditions, IPC can only be applied after the patient has become immobile. Immobility may then persist for weeks or even be permanent in such patients. DVTs may develop rapidly and cannot then be so effectively prevented by a treatment starting sometime after the initial period of paralysis and immobility. Clearly, we cannot test the effectiveness of IPC applied before stroke onset but it is a challenge to recruit patients into a RCT, with the need to collect informed consent, as early after the stroke as IPC might be applied if it was being used in routine clinical practice. For this reason we are trying to maximise the proportion of patients recruited on Day 0 or 1.

Another challenge in this trial is to optimise adherence to the IPC. To be a fair test of the device we need to achieve levels of adherence close to those which would be achieved if we knew that it was effective. However, in a randomised trial one has to achieve a balance between cajoling patients to persist with treatment which they might find uncomfortable whilst allowing them to stop the treatment without having to give a reason as stated in the consent procedure. Inevitably, adherence wanes over time because some patients find the IPC uncomfortable or staff became concerned by the condition of patient's skin. It is for this reason that we are planning to perform a secondary analysis of proximal DVTs up to 14 days.

Symptomatic DVTs affecting the popliteal or femoral veins, which occur within 30 days of randomisation, will be counted in the primary endpoint. A symptomatic DVT is defined for the purposes of this trial as a DVT confirmed on investigation with associated clinical features including leg swelling, pain, obvious erythema or a proven pulmonary embolus. Sometimes these features may not be recognised prior to a positive screening CDU but if present the DVT should be reported as symptomatic. We will distinguish those DVTs which were identified primarily on a screening CDU from those which were diagnosed clinically and confirmed on subsequent investigation since the detection of the latter symptomatic DVTs is not blinded and hence prone to ascertainment bias. A secondary analysis excluding these symptomatic DVTs identified before any CDU will also be performed. Inevitably, some patients will not survive to have routine CDUs and many of these will not have a detailed autopsy to establish whether they had a DVT or PE prior to death. However, it is possible that there will be an imbalance in the number of such deaths between the treatment groups especially if IPC is very effective in reducing the risk of fatal PE. Therefore, we will firstly present the numbers Alive with no DVT, Alive with DVT, Dead without prior DVT, and Missing. We will carry out our analyses in two ways: comparing DVT with no DVT with dead and missing patients excluded; and comparing DVT + dead with no DVT + missing.

We have tried to maximise the benefits to patients from participation in the trial. Our patient information leaflet provides important general information about stroke and its treatment. Our follow up with screening CDU allows us to detect asymptomatic DVTs which may, if undetected and untreated, lead to fatal pulmonary embolism. Lastly we feedback the information gleaned for the 6 month follow-up regarding patients' functional status, mood and pain back to the patients' general practitioners so that this information can inform their future management.

Limitations of the CLOTS trial 3 include the imperfect blinding of the ultrasonographers which could bias detection of our primary outcome, and our inability to blind patients and caregivers which might bias assessment of some of the secondary outcomes. Other limitations included: lack of screening logs; lack of central verification of negative scans or 100% source data verification, but we consider these are unlikely to introduce bias or alter the external validity of the results.

## Trial status

The first patient was enrolled into CLOTS 3 on the 8^th ^Dec 2008. We expect to complete recruitment of 2800 patients by the end of September 2012 and to report the results in the first half of 2013.

## Abbreviations

CDUS: Compression duplex ultrasound scan; DMC: Data monitoring committee; DVT: Deep vein thrombosis; GCP: Good clinical practice; GCS: Graduated compression stockings; IPC: Intermittent pneumatic compression; PE: Pulmonary embolism; QALY: Quality adjusted life years; RCT: Randomised controlled trial; TSC: Trial steering committee

## Competing interests

The authors have no financial or non-financial interests relevant to the submitted work except that Covidien, (Mansfield MA, USA) provide free supplies of their IPC devices and sleeves to hospitals participating in the trial. Neither Covidien or the funders of the study have any role in data collection, data storage, data analysis, drafting of reports, or the decision to publish although we will allow COVIDIEN to comment on the draft manuscripts describing our main results prior to final submission.

## Authors' contributions

MD is the chief investigator of the CLOTS 3 trial, wrote the protocol, was principle applicant for grant funding, manages the trial on a day to day basis and enrolls patients to one centre and carries out all 6 month telephone follow ups. He drafted this manuscript. PS, JR,JF are co-investigators, were involved in design, grant applications, are members of the steering committee and constructively commented on this manuscript. CG provides statistical support to the trial, is a member of the steering committee and commented on this manuscript. All authors read and approved the final manuscript.
